# Intercropping with legumes can reduce insect pest pressure but does not increase yield in winter oilseed rape ( *Brassica napus* L.)

**DOI:** 10.1002/ps.71020

**Published:** 2026-06-17

**Authors:** Chloé Aline Raderschall, Ola Lundin, Iman Raj Chongtham, Ann‐Charlotte Wallenhammar, Peter Anderson, Georg Carlsson, Mattias Christoffer Larsson

**Affiliations:** ^1^ Department of Plant Protection Biology The Swedish University of Agricultural Sciences Lomma Sweden; ^2^ Department of Biosystems and Technology The Swedish University of Agricultural Sciences Lomma Sweden; ^3^ Department of Ecology The Swedish University of Agricultural Sciences Uppsala Sweden; ^4^ Rural Economy and Agricultural Society, HS Konsult AB Örebro Sweden

**Keywords:** biological control, brassica pod midge, cabbage stem flea beetle, crop diversification, faba bean, IPM, pollen beetle

## Abstract

**BACKGROUND:**

Winter oilseed rape (WOSR, *Brassica napus*) is a major food, feed and fuel crop that heavily relies on pesticides and fertilisers to obtain maximum yield. We tested the potential of intercropping WOSR with legumes (frost‐sensitive spring faba bean, frost‐sensitive berseem clover or winter hardy pea) to reduce the abundance of and damage caused by the WOSR insect pest complex, enhance biocontrol and replace synthetic inputs with ecosystem functions. Each intercrop was tested under varying crop management practices (full or reduced fertilizer and pesticide input, WOSR row spacing and sowing time of legume crop relative to WOSR).

**RESULTS:**

WOSR – spring faba bean intercropping reduced the abundance of cabbage stem flea beetle larvae on average from 6.87 to 3.48 per plant compared to WOSR sole cropping, but a 10‐day sowing delay of the clover or spring faba bean intercrop increased cabbage stem flea beetle larvae per plant from 3.17 to 5.52 compared to when the crops were sown simultaneously. WOSR – spring faba bean intercropping also reduced pollen beetle bud damage by 34% (95% CI: 16%, 48%) compared to WOSR sole cropping. Parasitism rates of pollen beetle and cabbage seed weevil were not affected by intercropping or crop management. WOSR seed yield (kg ha^−1^) was 35% (95% CI: 13%, 51%) lower in WOSR‐spring faba bean intercropping compared to WOSR sole cropping, and 38% (95% CI: 26%, 47%) lower in low input compared to high input systems.

**CONCLUSION:**

Our results confirmed that intercropping WOSR with frost‐sensitive legumes can reduce insect pest pressure, but also highlighted a trade‐off with WOSR yield and the importance of legume sowing time in relation to WOSR. © 2026 The Author(s). *Pest Management Science* published by John Wiley & Sons Ltd on behalf of Society of Chemical Industry.

## Graphical abstract

Intercropping winter oilseed rape with spring faba bean reduced cabbage stem flea beetle and pollen beetle damage but also reduced crop yield. Sowing the intercrop simultaneously with winter oilseed rape was essential to maximize crop protection against cabbage stem flea beetles.

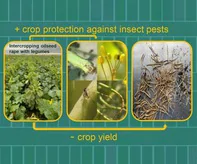

## 1 INTRODUCTION

Oilseed rape (*Brassica napus* L.) is a globally important crop for food, feed and fuel and a valuable break crop in cereal‐dominated cropping systems.^1^ There are both spring and winter forms of oilseed rape, with spring oilseed rape/canola being the dominant form grown in North America, and in Australia during winter, while the winter form is dominant in Europe.^2^, ^3^, ^4^ Winter oilseed rape (WOSR) in Europe is typically sown in August or September and harvested the following July–August.^5^ During this long cropping season, WOSR is prone to attacks by a series of insect pests at different crop stages,^6^ which cause significant yield losses across Europe.^7^ To date, synthetic chemical insecticides are still the predominant method used to protect the crop against these pests in Europe.^7^ WOSR also has a high dependency on nitrogen fertilizer inputs and herbicides to achieve optimal yields, which both have negative impacts on the environment.^8^, ^9^ Increasing restrictions on pesticide use and loss of efficacy of insecticides due to developing resistance threaten the profitability of oilseed rape production.,^10^, ^11^ Furthermore, insecticide applications in WOSR can be counterproductive,^12^ especially during flowering due to its deleterious effects on parasitoids, which are important biological control agents of several insect pests of WOSR.^13^, ^14^, ^15^ It is therefore necessary to design environmentally‐friendly WOSR cropping systems, where external inputs can be replaced with ecosystem functions yet high crop yields can be maintained.^16^

Crop diversification through intercropping, i.e., the simultaneous cultivation of two or more crop species in mixture, can offer several agronomic and environmental benefits and is a promising strategy to reduce the reliance on synthetic chemical inputs.^17^, ^18^ Intercrops can be integrated in cropping systems either as a service crop, where their primary purpose is to provide ecosystem services rather than marketable yield, or as a cash crop, where both crops are grown for production purposes.^19^, ^20^ Intercropping can enhance resource‐use efficiency by combining crop species with differential resource niches, strengthen the crops' competitiveness against weeds and reduce insect pest pressure.^21^, ^22^, ^23^, ^24^ Beneficial insects, such as predators and parasitoids of insect pests, can benefit from additional shelter and food resources that are provided by intercrops compared to sole crops, which can reduce pest populations by top‐down conservation biological control.^25^, ^26^, ^27^ Intercropping can also restrict arthropod pest colonization through bottom‐up effects, by impeding foraging physically^28^ or interfering with host localization behavior, for example by masking host plant odours.^29^ To date, most evidence of the suppressive effects of intercropping on insect pests has been collected from cereal‐legume intercropping systems, while oilseed rape‐legume intercropping has received less focus.^30^

Legumes can provide a range of ecosystem services when integrated into WOSR cropping systems. Frost‐sensitive legumes grown as service crops can increase the competition against weeds, suppress pest insects and reduce the reliance on nitrogen fertilizers thanks to the symbiotic fixation of atmospheric nitrogen in legume root nodules before they are killed by frost in winter.^19^, ^20^, ^31^ Field experiments that investigated WOSR insect pest responses to intercropping found that legume intercrops reduce insect pest abundance and crop damage of several key species and increase the pollen beetle parasitism rate.^32^, ^33^, ^34^, ^35^, ^36^ As a result of increased ecosystem services provision, intercropping with frost‐sensitive legumes can increase WOSR yield, or maintain yield with less fertilizer input,^37^ while overwintering grain legumes harvested as a second cash crop can increase total crop yield by enhancing resource‐use efficiency compared to when either crop is grown as a sole crop.^31^ Even though most studies show benefits of intercropping for pest control and yield, the practice is still not widely adopted by farmers due to perceived risks and challenges related to managing multiple crops at the same time, and a lack of evidence of the economic viability of intercropping.^23^, ^31^ To facilitate adoption of legume – oilseed rape intercropping systems, better understanding of the effects of intercropping on crop protection and crop yield under different management practices is needed to make more informed agronomic decisions.^31^

The major insect pests, which impact WOSR yield in northern Europe^6^ and which were studied here are the cabbage stem flea beetle (CSFB; *Psylliodes chrysocephala* Linnaeus: Coleoptera, Chrysomelidae), pollen beetle (*Brassicogethes aeneus* Fabricius: Coleoptera, Nitidulidae), cabbage seed weevil (*Ceutorhynchus obstrictus* Marsham: Coleoptera, Curculionidae) and brassica pod midge (*Dasineura brassicae* Winnertz: Diptera, Cecidomyiidae). CSFB cause damage both as adults and larvae; adults feed on cotyledons during establishment and larvae feed within plant stems and petioles throughout winter.^38^ When the first buds appear in spring, pollen beetles perforate flower buds during oviposition and feed on pollen, and feeding damage of both adults and larvae can lead to bud abscission, leaving podless stalks and thereby reducing yield.^6^, ^39^ Cabbage seed weevils immigrate into the fields during WOSR flowering and oviposit into the pods, where larvae feed on the maturing seeds.^6^, ^39^ Cabbage seed weevil oviposition holes also facilitate subsequent infestation by the brassica pod midge whose larvae feed on the inner walls of pods late in the season, causing premature pod shatter, leading to seed and thus yield loss.^39^, ^40^ WOSR insect pests are hosts to many, mostly Hymenopteran, parasitoids, which are important biological control agents.^41^ Yet the effect of intercropping on biological control by parasitoids has rarely been assessed.

Our objective was to determine if intercropping WOSR with different legume crops and crop management practices makes it possible to reduce fertilizer and pesticide inputs without causing yield losses compared to sole cropping of WOSR with standard input levels. For this, we assessed the effects of cropping system and crop management practices on insect pest abundance and crop damage, biological pest control by parasitoids and WOSR seed yield. Specifically, we compared frost‐sensitive with overwintering leguminous intercrops as well as WOSR cropping under different crop management (WOSR row distances, sowing dates of legumes relative to WOSR and fertilizer and pesticide input levels) to assess the benefits and trade‐offs regarding insect pest control and crop yield. We predicted that leguminous intercrops reduce insect pest damage in WOSR compared to WOSR sole cropping. We further expected that intercropping with frost‐sensitive legumes would increase WOSR yield at equal input level and maintain yield with reduced fertilizer and pesticide input, due to provision of several ecosystem services. We expected overwintering legumes to reduce WOSR yield independent of input level compared to WOSR sole cropping.

## 2 METHODS

### 2.1 Experimental design

We established a field experiment using a randomized block design replicated in four blocks and across two cropping seasons: 2022/2023 and 2023/2024. The experiment was placed on a different field each year, at the Swedish Infrastructure for Ecosystem Science (SITES) research station Lönnstorp (55°40′08.3″N 13°06′09.9″ E), southern Sweden, which is managed by the Swedish University of Agricultural Sciences. The field experiment included several treatments using 15 by 6 m plots (sown lengthwise), with WOSR as a sole crop and intercrops of WOSR and two frost‐sensitive legumes as service crops (berseem clover *Trifolium alexandrinum*), and spring faba bean *Vicia faba* minor), and two winter‐hardy grain legumes planned as second cash crops (winter pea *Pisum sativum* and winter faba bean), combined with a variety of establishment techniques (sowing dates, sowing densities, inter‐row distances) and input levels (Fig. 1, Table 1, Supporting Information, Table S1). Because our study was part of a larger project investigating the performance of WOSR‐legume intercropping systems for Swedish conditions, many different intercrop and management combinations were explored. For pragmatic reasons, i.e., resource limitation and practical relevance, certain treatment combinations were not tested, meaning that our experimental design was not fully balanced. For example, narrow row spacing was omitted for WOSR – winter pea intercropping because it would have required a lower WOSR sowing density to make room for both crops during summer and thus introduce yet one more factor in the experimental design. In cases where the experimental design is not balanced, conclusions for a factor are only valid for other treatment combinations, where this factor is represented.

**Figure 1:**
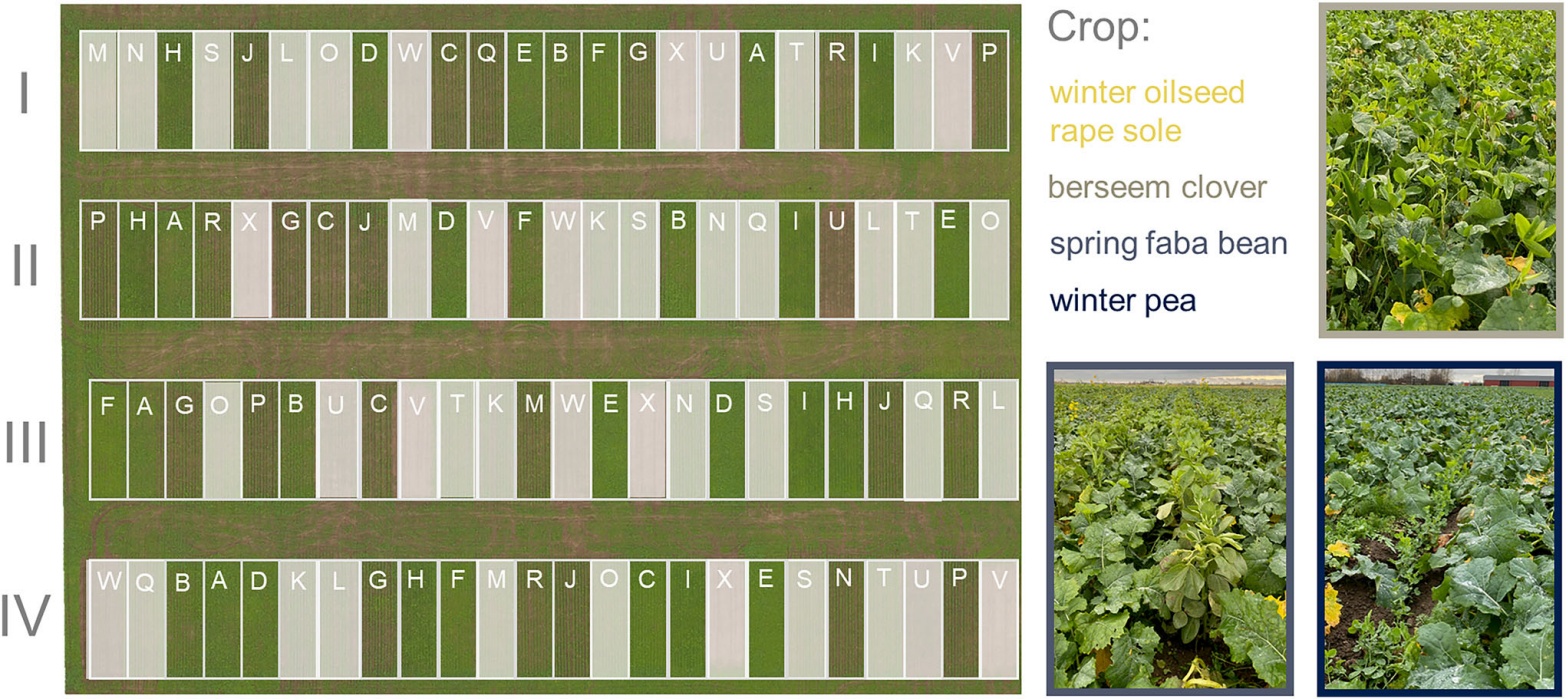
Drone image (2024) and schematic of the full experimental design, replicated in four blocks (I‐IV), where letters specify different treatment combinations that were randomised for each block and year, with winter oilseed rape (WOSR) sole crop, and WOSR rape intercropped with frost‐sensitive berseem clover, frost‐sensitive spring faba bean, and winter hardy pea (Table 1). Shaded plots indicate treatments that were part of the larger project but were excluded from this study (see Supporting Information, Table S1 for all treatment descriptions). Colors of the frames of the photos indicate legume intercrop (top right: berseem clover; bottom left spring faba bean; bottom right: winter pea). Photos of the intercrops were taken in November 2023. Drone photo: Ryan Davidson, other photos: Chloé Raderschall.

**Table 1:** Treatments that were part of the experimental design and where data for this study were collected

Treatment	Cropping system	Input level	WOSR row spacing	Sowing delay intercrop (days)	Sowing density intercrop (seeds/m^2^)
A	WOSR sole	high	narrow	no delay	not applicable
B	WOSR sole	high	wide	no delay	not applicable
C	WOSR sole	low	wide	no delay	not applicable
D	WOSR + clover	high	narrow	no delay	600
E	WOSR + clover	low	narrow	no delay	600
F	WOSR + clover	low	wide	no delay	600
G	WOSR + clover	low	wide	10	600
H	WOSR + spring faba	high	wide	no delay	30
I	WOSR + spring faba	low	wide	no delay	30
J	WOSR + spring faba	low	wide	10	30
P	WOSR + winter pea	high	wide	10	60
Q	WOSR + winter pea	low	wide	10	60
R	WOSR + winter pea	low	wide	42	60

*Note:* Winter oilseed rape (WOSR) sole cropping, intercropped with berseem clover, spring faba bean, winter faba bean or winter pea, and each winter hardy legume as sole crop. Insecticide, herbicide and fertilizer input levels, where high is equivalent to 100% of the recommended fertilizer and pesticide dose, and low is 75% of the recommended fertilizer dose and no pesticide use. WOSR row spacing, where narrow is 12,5 cm and wide is 50 cm. Sowing delay of the intercrop in days in relation to WOSR.

WOSR was sown on the 23 and 24 August 2022 and 2023, respectively. Sowing dates for the legumes varied relative to WOSR, depending on the treatment (Table 1). WOSR was sown with a row spacing of either 12.5 cm (narrow, 60 seeds per square meter) or 50 cm (wide, 50 seeds per square meter) depending on the treatment (Table 1). Further information on crop establishment and management is available in the Supplementary material (Text S1). Assessment of the winter pea treatments were only included in the 2023–2024 cropping season due to poor overwintering in the first year, and the treatments with winter faba bean were excluded from our assessment in both seasons due to poor overwintering. We used two input levels, high and low, whereby with high input levels, chemical and mechanical control of weeds, pests and diseases were performed when required and according to standard recommendations for yield optimization, while treatments under low input levels received no pesticides, but mechanical weed control was performed depending on the intercrop design (Supporting Information, Text S1, Table S2). Insecticide applications were only necessary once in high input treatments in autumn 2022 against CSFB (0.06 L ha^−1^ Nexide; Supporting Information, Table S2). Treatments under high input levels received the full rate of the recommended nitrogen fertilization for conventional WOSR management, while treatments under low input levels received 75% of the full rate (Supporting Information, Text S1). Sampling insect pest damage was restricted to within 1.5 m from plot edges to avoid interfering with harvest samples taken from plot centers.

### 2.2 Plant density

To evaluate crop establishment and overwintering, we estimated the WOSR plant density during stem elongation and early bud stage (BBCH 50–53^42^) in early April each year. For this we placed four 0.36 m^2^ quadrats per plot and counted the number of WOSR plants per quadrat.

### 2.3 Cabbage stem flea beetle (CSFB)

We quantified CSFB larval infestation in WOSR plants in early April, during stem elongation and early bud stage (BBCH 50–53). We cut 10 randomly selected plants at the base of the stem per plot and brought them to the laboratory. Plants were stored individually in plastic bags at 8 degrees Celsius for a maximum of 10 days, until they were processed. For each plant we noted the number of CSFB larvae by carefully dissecting plant stems^34^, ^43^ (Fig. 2(a)) and noted the plants' aboveground fresh biomass (g) and stem diameter (mm).

**Figure 2:**
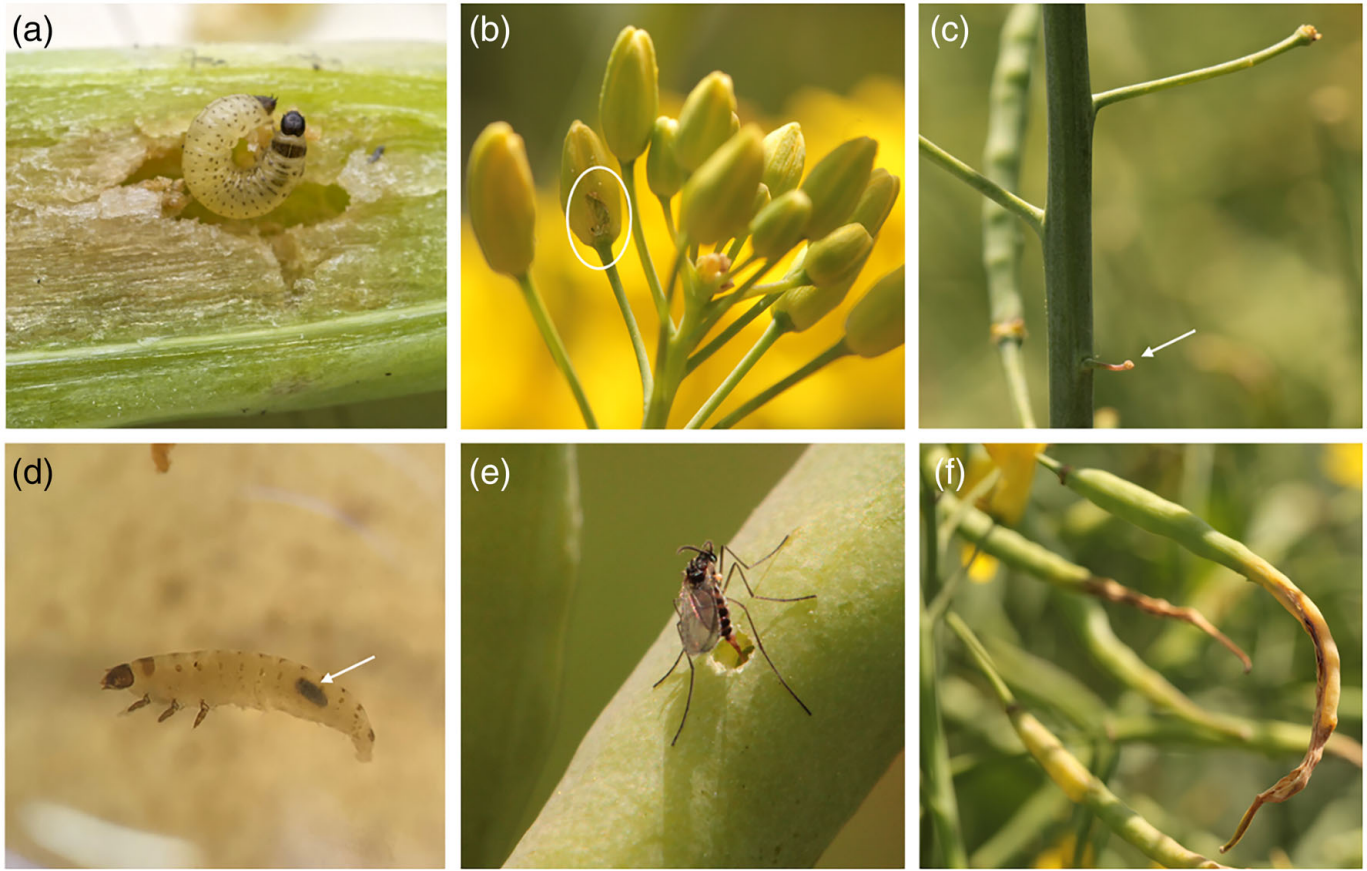
Oilseed rape insect pests and their crop damage: (a) cabbage stem flea beetle larvae inside stem, (b) pollen beetle feeding damage on bud (circled), (c) podless stalks following pollen beetle damage (white arrow), (d) pollen beetle larva with parasitoid egg (white arrow), (e) brassica pod midge ovipositing into a cabbage seed weevil larva or parasitoid exit hole, and (f) yellow distorted pod following brassica pod midge damage. Photos: Chloé Raderschall.

### 2.4 Pollen beetle and parasitism rate

We quantified pollen beetle damage at two crop growth stages. For the first assessment, which was only conducted in the second cropping season (2024), we quantified pollen beetle damage both during the green (BBCH 53–55) and the yellow (BBCH 57–59) bud stage. On 10 randomly selected plants within each plot we counted the number of buds on the main raceme that showed feeding damage (Fig. 2(b)).^44^

A second assessment was done during crop ripening (BBCH 73) in mid‐June in both years. We selected 10 random plants in each plot and estimated the proportion of podless stalks on the main raceme. We only considered stalks podless due to pollen beetle attack when their length was shorter than the length of stalks bearing pods (Fig. 2(c)).^44^

To quantify pollen beetle larvae and the parasitism rate of pollen beetle larvae by parasitoids, such as *Tersilochus heterocerus*, we deployed two plastic trays (surface area 735 cm^2^ each) filled with water and odorless detergent per plot during WOSR flowering in May and June (BBCH 61–69).^45^ We bound two plants together above each tray.^46^ We did this in three treatments in 2023 (A, E, I) and five treatments in 2024 (A, E, I, P, Q, Table 1). Trays were emptied and refilled weekly for 3 weeks in 2023 and 2 weeks in 2024. We counted all pollen beetle larvae and determined parasitism rates by dissecting all larvae and counting those larvae as parasitized, which had one or more parasitoid egg inside (Fig. 2(d)).^45^

### 2.5 Cabbage seed weevil and parasitism rate

Cabbage seed weevil infestation and parasitism rate was only assessed in the second cropping season (2024). We collected 10 pods (five from the main raceme and five from the third side raceme) from five randomly selected plants per plot in early July when pods had reached their final size and started to ripen (BBCH 80–81).^47^ We avoided taking pods that were already heavily deformed as a consequence of pod midge infestation, as this would have made it difficult to accurately assess the rate of seed weevil infestation and parasitism based on the presence of exit holes. In the laboratory, pods from the same plant were kept in paper bags in a climate chamber (25 °C, 50% humidity and 12 h light cycle) for 8 months to allow parasitoids to develop and emerge. Each pod was later examined under the light microscope for seed weevil or parasitoid exit holes (Fig. 2(e)) and all pods were opened to detect dead weevil larvae and parasitoids that failed to emerge. Seed weevil infestation was calculated as the number of exit holes from both seed weevil larvae and parasitoids, plus the number of dead weevil larvae and parasitoids that we found inside pods. The parasitism rate was calculated as the number of parasitoid exit holes on the pods plus dead parasitoids inside pods, divided by the total number of exit holes.^48^

### 2.6 Brassica pod midge

To quantify the damage caused by brassica pod midge, we used the same 10 randomly selected plants as we used to assess podless stalks (2.4) in each plot during crop ripening (BBCH 73) and estimated the proportion of yellow, swollen and distorted pods on the main raceme (Fig. 2(f)).^49^ Pods with these characteristic symptoms were also opened to confirm the presence of brassica pod midge larvae in both years.

### 2.7 Crop yield samples

The entire experiment was harvested on 30 July 2023 and 5 August 2024, using an experimental combine harvester, which collected and weighed the seed yield from a previously undisturbed central 24–30 m^2^ area in each plot (2 by 12 in 2023, 2 by 15 m in 2024). A sub‐sample of the harvested seeds from each plot (300–500 g) was dried (105 °C), cleaned from debris, weed seeds and from any legume seeds occurring in the intercrop treatments, and weighed to calculate the WOSR seed yield at 9% water content. Seed yield from intercropped winter pea in 2024 was collected but not further processed and analyzed for this experiment. We excluded data from nine plots in 2023 and five plots in 2024 due to mechanical problems with the weighing or during harvesting.

### 2.8 Statistical analysis

Statistical analyses were done using generalized linear mixed effects models in R^50^ version 4.4.1 for Windows using the glmmTMB function (package: glmmTMB^51^). Response variables were the number of plants per m^2^, plant fresh biomass (g), plant stem diameter (mm), CSFB larvae per plant, the number of buds damaged by pollen beetles per main raceme, proportion of podless stalks due to pollen beetles, number of pollen beetle larvae and larval parasitism rate, seed weevil infestation and seed weevil parasitism rate, proportion of pods damaged by pod midge and crop yield (kg per ha). All response variables were averaged per plot for the analyses. All models contained the explanatory variables cropping system (factor, four levels), input level (factor, two levels), WOSR row spacing (factor, two levels) and relative sowing time of legume intercrops (factor, three levels), except for the number of pollen beetle larvae and larval parasitism rate, where explanatory factors were only cropping system and input, because fewer treatments were sampled. We also included the two‐way interaction between cropping system and input level in all models as they were the only two fully crossed treatments in the experiment, with each input level being represented in all cropping systems. As random effects we added block (factor, four levels) nested within year (factor, two levels). For pollen beetle bud damage and seed weevil infestation and parasitism rate, which only was assessed in 2024, only block was included as the random effect. We chose residual distributions that resulted in the best model fits; Gaussian or gamma distributions for the averaged non‐integer count, weight and diameter data and beta regressions for the proportion data (Table 2). The amount of variance that contributed to a sample by the different treatment factors was analyzed with a type 2 ANOVA to account for unbalanced data^52^ (package: car^53^). We visually examined the residuals of each model to assure that model assumptions were met (package: DHARMa.^54^ The 95% confidence intervals were extracted using the emmeans function (package: emmeans^55^) and results plotted using ggplot2 (package ggplot2^56^ and viridis).^57^

**Table 2:** Statistical test results for the effect of cropping system (winter oilseed rape, WOSR sole crop, or intercropped with either berseem clover, spring faba bean or winter pea), input level (input high or low), WOSR row spacing (12.5 cm or 50 cm), sowing delay of the legume intercrop (no delay, 10 days or 6 weeks) and the two‐way interaction of cropping system and management intensity on the number of plants per m^2^, cabbage stem flea beetle larvae (CSFB) per plant, number of buds damaged by pollen beetles per main raceme, proportion of podless stalks per main raceme as a consequence of pollen beetle feeding, number of pollen beetle larvae and larval parasitism rate, proportion of pods infested with cabbage seed weevils and cabbage seed weevil parasitism rate, proportion of pods damaged by brassica pod midge per main raceme and WOSR seed yield (kg ha^−1^)

	Cropping system		Input		Row spacing		Sowing delay		Cropping system: input		Error distribution	N observations
	Chi Sq	*P*	Chi Sq	*P*	Chi Sq	*P*	Chi Sq	*P*	Chi Sq	*P*		
Plants per m^2^	**12.49**	**0.0059**	<0.01	0.96	2.39	0.12	2.71	0.26	1.58	0.67	gamma	88
CSFB larva	**10.83**	**0.013**	**7.32**	**0.0068**	0.95	0.33	**8.60**	**0.014**	6.59	0.086	gamma	88
Pollen beetle bud damage	**20.68**	**<0.001**	0.020	0.89	0.31	0.58	3.0	0.23	1.83	0.61	gamma	52
Podless stalks	2.19	0.53	<0.01	0.95	0.54	0.46	2.34	0.31	1.96	0.58	beta binomial	92
Pollen beetle larvae	1.82	0.61	0.068	0.80	‐	‐	‐	‐	‐	‐	gaussian	32
Pollen beetle parasitim rate	0.95	0.81	0.38	0.54	‐	‐	‐	‐	‐	‐	beta binomial	32
Seed weevil infestation	5.45	0.14	0.16	0.69	0.50	0.48	4.60	0.10	1.84	0.61	gamma	52
Seed weevil parasitism rate	1.86	0.60	0.91	0.34	0.33	0.56	5.74	0.057	6.82	0.078	beta binomial	52
Pod midge damaged pods	**9.78**	**0.021**	2.48	0.12	0.035	0.85	1.87	0.39	2.09	0.55	beta binomial	92
WOSR yield (kg ha^−1^)	**19.64**	**<0.001**	**42.07**	**<0.001**	0.39	0.53	1.28	0.53	7.20	0.066	gaussian	80

*Note:* Shown for each tested variable are the chi‐square (Chi Sq) and *P*‐values (P). Degrees of freedom for all models were: cropping system = 3, input = 1, row spacing =1, sowing delay =2, Cropping system: input = 3. For each model we also provide the error distribution used and the number of observations analyzed by each model. Statistically significant (*P* < 0.05) results are indicated in bold.

## 3 RESULTS

### 3.1 WOSR plant density

WOSR plant density was on average 30.8 (std.dev.: 6.6) plants per m^2^ but differed depending on the cropping system (Table 2, Supporting Information, Fig. S1). WOSR sole cropping had 21% (95% CI: 2%, 42%) higher plant density than WOSR – spring faba bean intercropping, while WOSR – winter pea and berseem clover intercropping had intermediate plant densities that were not significantly different from the others.

### 3.2 Cabbage stem flea beetle larvae, plant biomass and plant stem diameter

The average number of CSFB larva per WOSR plant was 4.3 (std.dev.: 2.7) and differed depending on the cropping system, sowing delay of the intercrop and management intensity. There were 98% (95% CI: 22%, 219%) more CSFB larva per plant in plots with WOSR sole cropping than in WOSR – spring faba bean intercropping (Table 2, Fig. 3(a)). There were 43% (95% CI: 10%, 63%) fewer CSFB larvae per WOSR plant when WOSR and the clover or faba bean intercrop were sown simultaneously compared to when the intercrop was sown 10 days after WOSR (Table 2, Fig. 3(b)). The high input treatment also had 25% (95% CI: 5%, 46%) fewer CSFB larvae than the low input treatment (Table 2, Fig. 3(c)). There was a tendency that the average number of CSFB larva per WOSR plant was explained by an interaction between cropping system and input level, although there were no significant differences among cropping systems within each input level (Table 2, Supporting Information, Fig. S2a). WOSR plant fresh biomass (g) also differed among cropping system and sowing delay of the intercrop. WOSR plant biomass was 92% (95% CI: 31%, 182%) higher in WOSR sole cropping compared to WOSR – spring faba bean intercropping (Table 2, Supporting Information, Fig. S2b) and 36% (95% CI: 10%, 55%) lower when WOSR and the clover or spring faba bean intercrop were sown at the same time compared to when the intercrop was sown 10 days after WOSR (Table 2, Supporting Information, Fig. S2c). There was also a tendency towards WOSR plants having a higher biomass when row spacing was wider (Table 2, Supporting Information, Fig. S2d).

**Figure 3:**
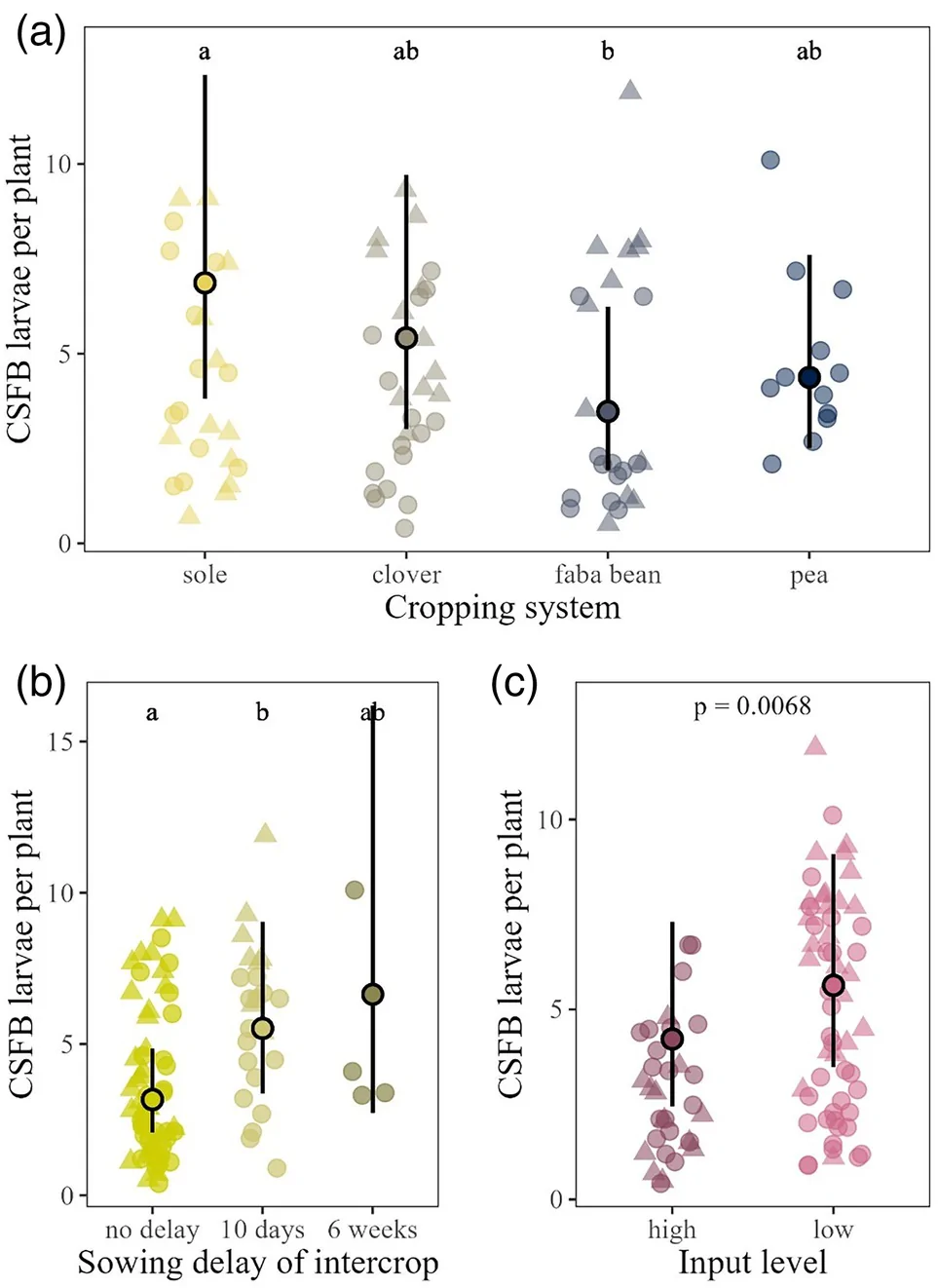
Average number of cabbage stem flea beetle (CSFB) larvae per winter oilseed rape (WOSR) plant in April over 2 years, 2023 (triangles) and 2024 (dots) in respect to (a) cropping system (WOSR sole crop, or intercropped with either berseem clover, spring faba bean or winter pea), (b) sowing delay of the intercrop in respect to WOSR, and (c) management intensity. Shown are data points (shaded) estimated marginal means and 95% confidence intervals. Treatments that do not share any letter are significantly different. (*P* < 0.05) from each other based on pairwise contrasts with Tukey correction. The *P*‐value for c), where only two treatments are compared, refers to the outcome of the type II Chi‐square test.

Similarly, WOSR stem diameter was affected by sowing delay of the intercrop, with WOSR stem diameters being 16% (95% CI: 6%, 25%) lower in treatments, where WOSR and the clover or spring faba bean intercrop were sown at the same time than when the intercrop was sown 10 days after WOSR (Table 2, Supporting Information, Fig. S2e). There was also a tendency that WOSR stem diameter differed among cropping systems, with stem diameters being higher in WOSR sole cropping compared to any legume intercropping system (Table 2, Supporting Information, Fig. S2f).

### 3.3 Pollen beetle and parasitism rate

There was an average of 4.8 (std.dev.: 1.2) damaged buds on WOSR main racemes as a consequence of pollen beetle feeding, and the number of damaged buds differed depending on the cropping system. WOSR – spring faba bean intercropping reduced the number of damaged buds by 34% (95% CI: 16%, 48%) compared to WOSR sole cropping (Table 2, Fig. 4).

**Figure 4:**
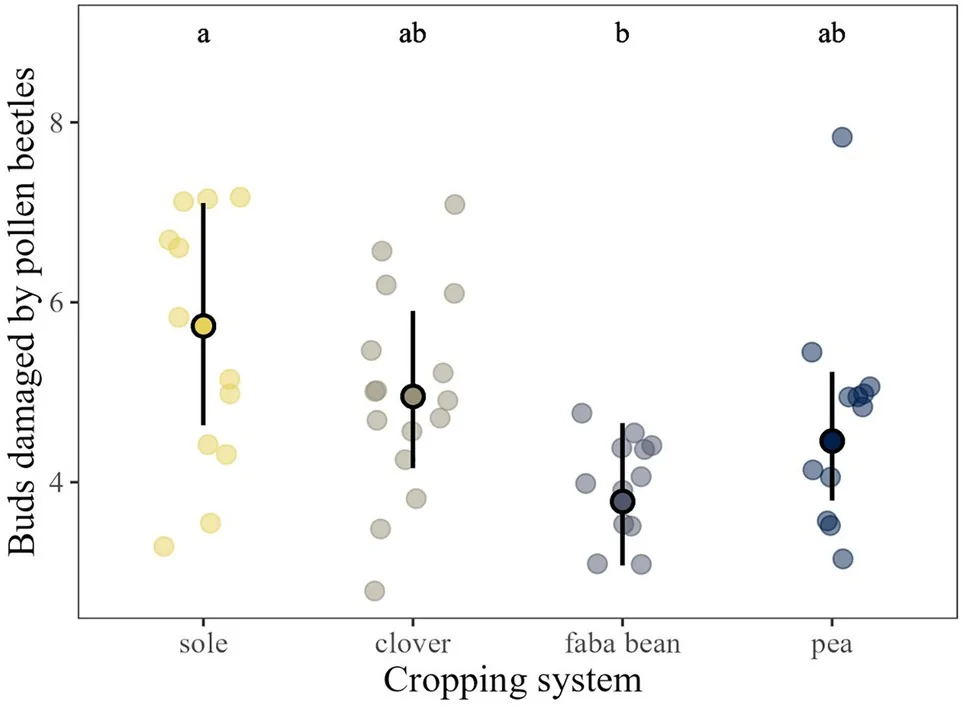
The average number of buds damaged by pollen beetles per main raceme in respect to cropping system (winter oilseed rape, WOSR sole crop, or WOSR intercropped with either berseem clover, spring faba bean or winter pea) in 2024. Shown are data points (shaded), estimated marginal means and 95% confidence intervals. Treatments that do not share any letter are significantly different. (*P* < 0.05) from each other based on pairwise contrasts with Tukey correction.

The average proportion of podless stalks per main raceme caused by pollen beetle feeding was 0.28 (std.dev.: 0.078) and was not affected by any treatment (Table 2).

There was an average number of 10.7 (std.dev.: 6.3) pollen beetle larvae per tray and the average parasitism rate was 0.68 (std.dev.: 0.14). Neither the number of pollen beetle larvae nor the parasitism rate was affected by any treatment (Table 2).

### 3.4 Cabbage seed weevil and parasitism rate

The average number of seed weevils infesting pods per plant was 4.3 (std. dev.: 1.4) and it was not affected by cropping system or management practices (Table 2). On average, the seed weevil larval parasitism rate was 0.69 (std. dev.: 1.8). There was a tendency that the parasitism rate was affected by sowing delay and an interaction between cropping system and input level, but post‐hoc tests did not show any difference among treatments (Table 2, Supporting Information, Fig. S3a,b).

### 3.5 Brassica pod midge

The average proportion of pods damaged by brassica pod midge per main raceme was 0.24 (std.dev.:0.087) and varied depending on the cropping system. WOSR – spring faba bean intercropping had a 42% (odds ratio 95% CI: 0.275, 0.862) lower proportion of pods damaged by brassica pod midge than WOSR – winter pea intercropping (Table 2, Fig. 5).

**Figure 5:**
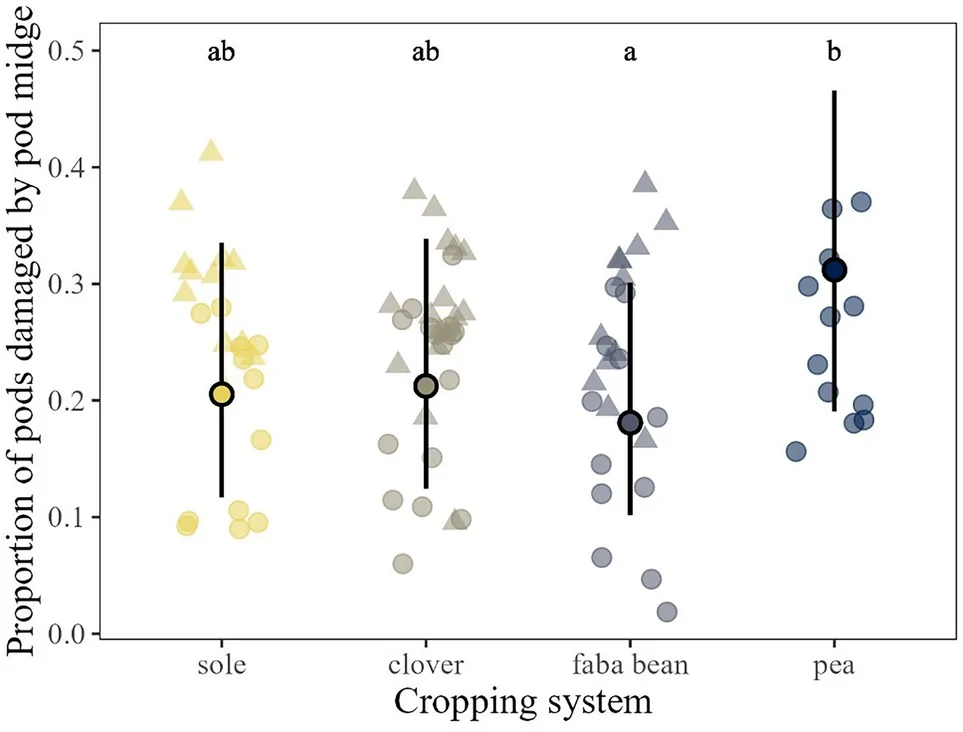
Proportion of pods damaged by brassica pod midge per main raceme in respect to cropping system (winter oilseed rape sole crop, or intercropped with either berseem clover, spring faba bean or winter pea) over 2 years, 2023 (triangles) and 2024 (circles). Shown are data points (shaded), estimated marginal means and 95% confidence intervals. Treatments that do not share any letter are significantly different. (*P* < 0.05) from each other based on pairwise contrasts with Tukey correction.

### 3.6 WOSR yield

The average WOSR seed yield at a seed moisture of 9% was 1454 kg (std.dev.: 670) per hectare and was influenced by both cropping system and input level. WOSR yield was 35% (95% CI: 13%, 51%) lower in WOSR – spring faba bean intercropping compared to WOSR sole cropping (Table 2, Fig. 6(a)). WOSR crop yield was also 38% (95% CI: 26%, 47%) lower in low input treatments compared to high input treatments (Table 2, Fig. 6(b)). There was a tendency that WOSR yield was explained by an interaction between cropping system and input level, with WOSR yield varying among treatments at high input levels but not low input levels (Table 2, Supporting Information, Fig. S4).

**Figure 6:**
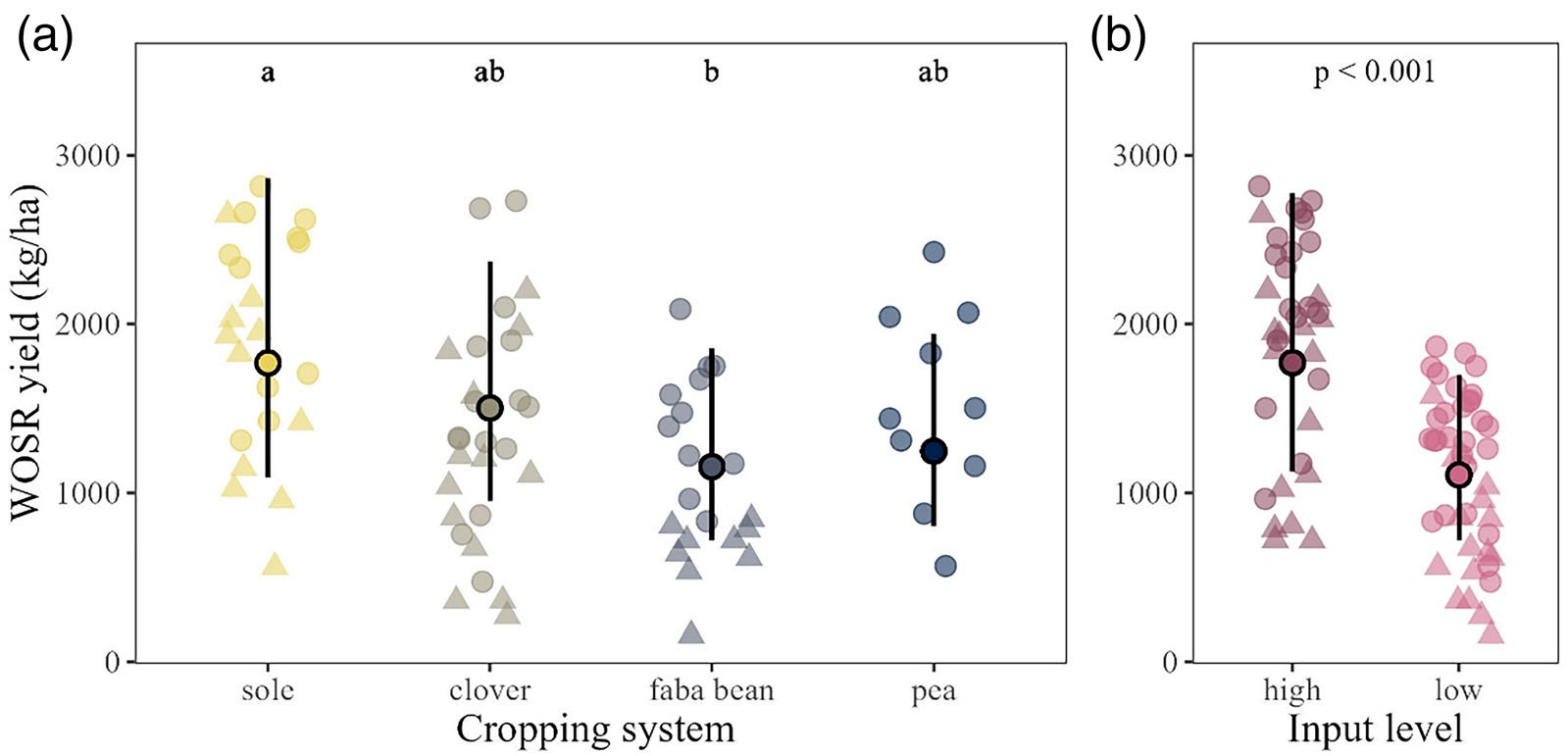
The average winter oilseed rape (WOSR) seed yield (kg per ha, 9% moisture content) in respect to (a) cropping system (WOSR sole crop, or intercropped with either berseem clover, spring faba bean or winter pea) and (b) input level (high, low) over 2 years, 2023 (triangles) and 2024 (circles). Shown are data points (shaded), estimated marginal means and 95% confidence intervals. Treatments that do not share any letter are significantly different. (*P* < 0.05) from each other based on pairwise contrasts with Tukey correction.

## 4 DISCUSSION

In a replicated field trial over two growing seasons in Sweden, we showed that intercropping WOSR with spring faba bean has the potential to enhance crop protection against insect pests, but not WOSR crop yield. Instead, WOSR – spring faba bean intercropping reduced crop yield compared with WOSR sole cropping. Intercropping with berseem clover and winter pea had no effects on insect pest abundance, crop damage and WOSR yield.

The mean number of CSFB larvae per plant decreased from 6.87 to 3.48 when WOSR was intercropped with spring faba bean compared to WOSR sole cropping, bringing larval infestation below the threshold of five larvae per plant, at which significant crop yield losses are expected.^58^ Our result is in line with findings from field experiments where intercropping WOSR with frost sensitive legumes as compared to WOSR sole cropping reduced CSFB larval infestation,^35^, ^59^ and the numbers of CSFB adults immigrating in autumn.^33^ Interestingly, our results showed that sowing the intercrop simultaneously with WOSR is essential for pest control. Already a short sowing delay of 10 days between the clover or spring faba bean intercrop and WOSR sowing increased the number of CSFB from 3.17 to 5.52 larvae per plant compared to when the crops were sown simultaneously. Thus, not only intercrop species, but also sowing time are important measures to consider to maximize the efficacy of intercropping for crop protection. To our knowledge the effect of sowing time of the intercrop on insect pest abundance in WOSR has not been shown before. Since we did not monitor the immigration time of CSFB to the field and we do not know at what growth stage the intercrop was, at the time of immigration, it is not possible to determine what mechanisms were responsible for driving the observed increase in CSFB larvae following an intercrop sowing delay. However, it is likely that the reduction of CSFB larvae in WOSR‐spring faba bean cropping systems and in treatments where WOSR and intercrops were sown at the same time was mediated by WOSR plant biomass and stem diameter, both of which were lower in treatments with fewer CSFB larvae, i.e., WOSR ‐ spring faba bean intercropping and no sowing delay treatments. This hypothesis is supported by evidence from England and Germany, where companion plants reduced WOSR plant biomass, and lower WOSR biomass was in turn related to lower CSFB larval abundance.^59^ The effect of input intensity on CSFB larval abundance was likely driven by a single insecticide application in the high input treatment in 2022 (Supporting Information, Table S2), which reduced the number of larvae per plant in high input treatments.

Intercropping WOSR with spring faba bean also reduced bud damage caused by pollen beetle feeding in spring, but there was no effect on the proportion of podless stalks as a consequence of pollen beetle feeding. In line with our result, a field experiment from Switzerland recorded fewer pollen beetle adults per plant in WOSR – spring faba bean intercropping compared to WOSR sole cropping, whereas crop damage from pollen beetles was not assessed.^33^ Assessing pollen beetle bud damage is considered a more accurate indication of crop yield loss related to pollen beetles than counting adult beetles, due to high temporal variation of adult numbers depending on weather conditions,^44^ and because adult pollen beetle feeding intensity depends on temperature.^60^ We found, however, no effect of cropping system on the proportion of podless stalks caused by pollen beetles, which is usually correlated with pollen beetle feeding damage in buds.^44^ It could be that not all bud feeding damage was detrimental to pod development so that the effects of cropping system on the proportion of podless stalks was less pronounced, or that plants were able to compensate for the bud damage.

The proportion of WOSR pods damaged by brassica pod midge was affected by cropping system, where WOSR – spring faba bean intercropping showed lower damage compared to WOSR – winter pea intercropping, whereas WOSR sole cropping and WOSR – berseem clover intercropping had intermediate damage levels. Our result contrasts with an earlier Swedish field experiment, where brassica pod midge damage was highest in WOSR – berseem clover intercropping, and lowest in WOSR – winter pea intercropping.^35^ Considering that brassica pod midge host plant selection relies on olfactory and visual cues of the host plant,^40^ it would be expected that an overwintering intercrop would be more effective at interfering with host selection than a frost‐sensitive intercrop, which is no longer present at the time of oviposition. As such, it could be that the varying effects of winter pea intercropping could be explained by differences in intercrop establishment and overwintering success, which in turn affect cues and quality of the WOSR host plant for brassica pod midge.

The biological control of insect pests carried out by parasitoids was not affected by the cropping system or crop management. This contrasts with earlier findings, where intercropping with winter barley, winter rye and winter pea increased the parasitism rate of pollen beetles compared to WOSR sole cropping.^36^ It is possible that our treatment plots were too small and too close together to depict differences in parasitism. In addition, we also found no differences in pollen beetle and cabbage seed weevil larval abundance among treatments, suggesting that host availability was equal across all treatments. Notably, a high proportion of both pollen beetle and cabbage seed weevil larvae were parasitized independent of the experimental treatment, confirming the importance of parasitoids for conservation biological control in WOSR.^45^, ^48^, ^60^

The seed yield of WOSR (kg ha^−1^) was 35% lower in WOSR – spring faba bean intercropping compared to WOSR sole cropping, while intercropping WOSR with berseem clover and winter pea had intermediate yield levels, and yield was 38% lower in low input systems compared to high input systems. The yield loss associated with WOSR‐spring faba bean intercropping contrasts with earlier research. In French field experiments, WOSR yield was higher when intercropped with spring faba bean compared to sole cropping under recommended fertilization rates, and yield levels were maintained or even increased when nitrogen inputs were reduced by 30–40 kg ha^−1^.^32^, ^37^ It could be that weed‐crop competition and subsequent WOSR yield loss was higher in intercropping systems compared to WOSR sole cropping, as legume intercropping restricted possibilities for both herbicide use in high input treatments and row hoeing in low input treatments (Supporting Information, Table S2). It is also possible that the shorter autumn and lower light irradiation at the latitudes of our study site limited the contribution of biologically fixed nitrogen from legumes, and made competition between WOSR and intercrops more critical. The biomass growth of spring faba bean in autumn was beneficial for crop protection against pests, but simultaneously might have increased competition with WOSR, or impacted WOSR physically, i.e., by lodging, when the faba bean plants died from frost, causing yield losses.^20^, ^61^ We chose relatively high densities of the intercrops (Table 1, Supporting Information, S2), possibly higher than what is common in warmer climates, because the time for them to establish is quite short and to maximize the chances of winter hardy legumes surviving the winter. Since excessive intercrop biomass increases the risk of competition with cash crops,^20^ reducing the spring faba bean sowing density could be a potential solution to reduce competition while maintaining crop protection benefits. When intercropping WOSR, trade‐offs are common between providing ecosystem services such as pest control and causing disservices such a yield loss due to competition.^61^ In line with this, intercropping WOSR with berseem clover or winter pea did not cause WOSR yield losses in our field experiment (and in the case of winter pea produced a second harvestable crop yield), but also did not improve pest control. A better understanding of which intercrop traits cause services and disservices will be important to counteract or adapt to disservices with crop management.^19^, ^61^ The relatively low average WOSR seed yield in our experiment compared to the regions' (Skåne) average in conventional production over the past 15 years of 3636 kg ha^−1^ ^62^ is attributed to a harsh frost in spring 2023, which reduced WOSR in the whole region to an average of 2330 kg ha^−1^ ^62^ and the low pesticide and fertilizer inputs in some of our treatments. It is also likely that the small experimental plots were more susceptible to herbivore attacks and thus had lower yields than large commercial fields in the same region. An interesting trend in our case deserving further study was that the pest regulation – yield trade‐off from intercropping WOSR with legumes was less strong at low input levels.

Taken together, the results from our field experiment show that the effects of WOSR – legume intercropping on insect pest regulation and crop yield depend on legume species and intercrop sowing time relative to WOSR. In general, the influence of cropping environment and crop management practices is both a challenge and an obstacle for the practical implementation of ecological intensification practices that aim to replace inputs with ecosystem functions.^16^, ^63^ An improved theoretical understanding of such context dependencies coupled with further experimentation is therefore needed for ecosystem services to be successful in replacing inputs in practice by intercropping WOSR with legumes.

## CONFLICT OF INTEREST

The authors declare that they have no known competing financial interests or personal relationships that could have appeared to influence the work reported in this paper.

## AUTHOR CONTRIBUTIONS

Chloé A. Raderschall: Conceptualization, Methodology, Investigation, Validation, Formal analysis, Supervision, Visualization, Writing – original draft, Writing – review & editing. Ola Lundin: Conceptualization, Methodology, Writing – review & editing. I. Raj Chongtham: Conceptualization, Methodology. Ann‐Charlotte Wallenhammar: Conceptualization, Methodology, Writing – review & editing. Peter Anderson: Conceptualization, Funding acquisition, Project Administration, Writing – review & editing. Georg Carlsson: Conceptualization, Methodology, Funding acquisition, Project Administration, Writing – review & editing. Mattias C. Larsson: Conceptualization, Investigation, Project Administration, Supervision, Writing – review & editing.

## Supporting information


**Text S1.** Crop management details.
**Table S1.** Treatments that were part of the full experimental design. Winter oilseed rape (WOSR) sole cropping, intercropped with berseem clover, spring faba bean, winter faba bean or winter pea, and each winter hardy legume as sole crop. Insecticide, herbicide and fertilizer input levels, where high is equivalent to 100% of the recommended fertilizer and pesticide dose, and low is 75% of the recommended fertilizer dose and no pesticide use. WOSR row spacing, where narrow is 12,5 cm and wide is 50 cm. Sowing delay of the intercrop in days in relation to WOSR. Treatments that are indicated with a ‡ symbol were part of the design but not included in this study.
**Table S2.** Details of insecticide use, herbicide use and mechanical weed control (date, rate and compound or type of mechanical weed control) in each treatment and year of the field experiment. 1 gamma‐cyhalothrin 60 g L^−1^. 2 cycloxidim 100 g L^−1^. 3 halauxifen‐methyl 10 g L^−1^ + picloram 48 g L^−1^. 4 propyzamide 400 g L^−1^. 5 clopyralid 720 g kg^−1^.
**Fig. S1.** The average number of winter oilseed rape (WOSR) plants per m^2^ in April over 2 years, 2023 (triangles) and 2024 (dots) in respect to cropping system (oilseed rape sole crop, or intercropped with either berseem clover, spring faba bean or winter pea. Shown are data points (shaded), estimated marginal means and 95% confidence intervals. Different letters denote treatments that are significantly different from each other (*P* < 0.05) based on pairwise contrasts with Tukey correction.
**Fig. S2.** Cabbage stem flea beetle larvae per plant, winter oilseed rape (WOSR) fresh plant weight and stem diameter in April over 2 years, 2023 (triangles) and 2024 (dots). (a) Average number of cabbage stem flea beetle (CSFB) larvae per WOSR plant in respect to cropping system (WOSR sole crop, or intercropped with either berseem clover, spring faba bean or winter pea) and input level (high, low). WOSR fresh plant weight (g) in respect to (b) cropping system, (c) sowing delay of the intercrop (no delay, 10 days or 6 weeks delay) and (d) WOSR row distance (narrow: 12.5 cm; wide: 50 cm). WOSR stem diameter (mm) in respect to (e) sowing delay of the intercrop and (f) cropping system. Shown are data points (shaded), estimated marginal means and 95% confidence intervals. Different letters denote treatments that are significantly different from each other (*P* < 0.05) based on pairwise contrasts with Tukey correction.
**Fig. S3.** The rate of parasitism of cabbage seed weevil larvae in 2024 in respect to (a) sowing delay of the intercrop and (b) and interaction between input level and cropping system (winter oilseed rape sole crop, or intercropped with either berseem clover, spring faba bean or winter pea) and input level (high, low). Shown are data points (shaded), estimated marginal means and 95% confidence intervals. Different letters denote treatments that are significantly different from each other (*P* < 0.05) based on pairwise contrasts with Tukey correction.
**Fig. S4.** The average winter oilseed rape (WOSR) seed yield (kg per ha, 9% moisture content) in respect to cropping system (WOSR sole crop, or intercropped with either berseem clover, spring faba bean or winter pea) and input level (high, low) over 2 years, 2023 (triangles) and 2024 (circles). Shown are data points (shaded), estimated marginal means and 95% confidence intervals. Different letters denote treatments that are significantly different from each other (*P* < 0.05) based on pairwise contrasts with Tukey correction.

## Data Availability

The data that support the findings of this study will be openly available via the Swedish National Data repository (SND) at https://doi.org/10.5878/esfa-pq39.
