# Supplementary material for: Intercropping with legumes can reduce insect pest pressure but does not increase yield in winter oilseed rape ( Brassica napus L.)
Source: Pest Manag Sci. 2026 Jun 17;82(11):10705–15. doi: 10.1002/ps.71020 (PMC13665645; doi:10.1002/ps.71020)
Supplement: Supplementary file 1 [file PS-82-10705-s001.docx]

**Supplementary material for:**

**Intercropping with legumes can reduce insect pest pressure but does not increase yield in winter oilseed rape (*Brassica napus* L.)**

Chloé Aline Raderschall^1,2,^*, Ola Lundin^3^, Iman Raj Chongtham^2^, Ann-Charlotte Wallenhammar^4^, Peter Anderson^1^, Georg Carlsson^2,^ ^†^, Mattias Christoffer Larsson^1,^ ^†^

^1^Department of Plant Protection Biology, The Swedish University of Agricultural Sciences, Box 190, 234 22 Lomma, Sweden

^2^Department of Biosystems and Technology, The Swedish University of Agricultural Sciences, Box 190, 234 22 Lomma, Sweden

^3^Department of Ecology, The Swedish University of Agricultural Sciences, Box 7044,

750 07 Uppsala, Sweden

^4^ Rural Economy and Agricultural Society, HS Konsult AB, Gamla vägen 5G, 702 27 Örebro, Sweden

*Correspondence to: chloe.raderschall@slu.se

†Joint senior authorship

**Text S1.** *Crop management details*
The experiment was placed in fields with conventionally managed WOSR, although in 2022/2023 the field was only slightly larger than the experiment. In both years, the experimental area was ploughed and harrowed two times before any crops were sown. In 2022/2023, winter oilseed rape (WOSR) was sown on 2022-08-23 together with berseem clover and spring faba bean in treatments with no sowing delay. Berseem clover and spring faba bean was sown on 2022-09-07 in treatments assigned a 10 day delay (i.e.,15 days actual delay). WOSR was harvested on 2023-07-30. In 2023/2024, WOSR, berseem clover and spring faba bean with no sowing delay was sown on 2023-08-24. Berseem clover, spring faba bean and winter peas in treatments with a 10 day sowing delay were sown on 2023-09-06. Winter pea treatments assigned a six week sowing delay were sown on 2023-09-28 (i.e., 35 days actual delay). Reasons why delayed sowing of the intercrop could be beneficial and was included as treatments here are 1) delaying intercrop sowing might give WOSR a headstart and improve its performance, 2) delayed sowing of the intercrop could be combined with soil disturbance to mechanically remove weeds and 3) winter faba bean and winter peas need a delayed sowing relative to WOSR to not grow too big before overwintering. Cultivars used were Crocodile (WOSR), Tim (berseem clover, 2022-2023) and Cleopatra (berseem clover, 2023-2024), Fanfare (spring faba bean), Augusta (winter faba bean) and Pandora (winter pea). WOSR sowing density was 60 and 50 seeds per square meter in the narrow and wide row spacing, respectively. Berseem clover was broadcast sown immediately before sowing WOSR or immediately before inter-row hoeing (depending on sowing date treatment), meaning that the clover seeds were mixed with the uppermost 1-2 cm soil by the WOSR sowing or inter-row hoeing operations. Spring faba bean was sown with a row distance of 50 cm and winter faba bean and winter pea were sown with a row distance of 12.5 cm as sole crops and 50 cm when sown as an intercrop. Treatments under high input levels received the full rate of the recommended nitrogen (N) fertilisation; 213 kg N per ha total; 40 kg applied as YaraMila Raps (Yara AB, Sweden) and 13 kg applied as YaraLiva Kalksalpeter (Yara AB, Sweden) in September and 160 kg applied as Suprasalpeter N27 (Yara AB, Sweden) in March, for conventional WOSR management, while treatments under low input levels received only 75% of the full rate (160 kg N per ha; 40 kg in September and 120 kg in March). Phosphorous (P = 11.5 kg per ha) and potassium (K= 23.5 kg per ha) were applied in autumn as part of the YaraMila Raps application and were the same across all WOSR treatments. Insecticide use, herbicide use and mechanical weed control operations in each treatment and year is presented in Table S2.

**Table S1** Treatments that were part of the full experimental design. Winter oilseed rape (WOSR) sole cropping, intercropped with berseem clover, spring faba bean, winter faba bean or winter pea, and each winter hardy legume as sole crop. Insecticide, herbicide and fertiliser input levels, where high is equivalent to 100% of the recommended fertiliser and pesticide dose, and low is 75% of the recommended fertiliser dose and no pesticide use. WOSR row spacing, where narrow is 12,5 cm and wide is 50 cm. Sowing delay of the intercrop in days in relation to WOSR. Treatments that are indicated with a ‡ symbol were part of the design but not included in this study.

| treatment | cropping system | input level | WOSR row spacing | sowing delay intercrop (days) | sowing density intercrop (seeds/m^2^) |
| --- | --- | --- | --- | --- | --- |
| A | WOSR sole | high | narrow | no delay | not applicable |
| B | WOSR sole | high | wide | no delay | not applicable |
| C | WOSR sole | low | wide | no delay | not applicable |
| D | WOSR + clover | high | narrow | no delay | 600 |
| E | WOSR + clover | low | narrow | no delay | 600 |
| F | WOSR + clover | low | wide | no delay | 600 |
| G | WOSR + clover | low | wide | 10 | 600 |
| H | WOSR + spring faba | high | wide | no delay | 30 |
| I | WOSR + spring faba | low | wide | no delay | 30 |
| J | WOSR + spring faba | low | wide | 10 | 30 |
| K‡ | WOSR + winter faba | high | wide | 10 | 30 |
| L‡ | WOSR + winter faba | low | wide | 10 | 30 |
| M‡ | WOSR + winter faba | low | wide | 42 | 30 |
| N‡ | WOSR + winter faba | low | wide | 10 | 15 |
| O‡ | WOSR + winter faba | low | wide | 42 | 15 |
| P | WOSR + winter pea | high | wide | 10 | 60 |
| Q | WOSR + winter pea | low | wide | 10 | 60 |
| R | WOSR + winter pea | low | wide | 42 | 60 |
| S‡ | WOSR + winter pea | low | wide | 10 | 30 |
| T‡ | WOSR + winter pea | low | wide | 42 | 30 |
| U‡ | winter faba sole | low | narrow | 10 | 60 |
| V‡ | winter faba sole | low | narrow | 42 | 60 |
| W‡ | winter pea sole | low | narrow | 10 | 120 |
| X‡ | winter pea sole | low | narrow | 42 | 120 |

**Table S2.** Details of insecticide use, herbicide use and mechanical weed control (date, rate and compound or type of mechanical weed control) in each treatment and year of the field experiment. 1 gamma-cyhalothrin 60g L-1. 2 cycloxidim 100 g L-1. 3 halauxifen-methyl 10 g L-1 + picloram 48 g L-1. 4 propyzamide 400 g L-1. 5 clopyralid 720 g kg-1.

| 2022-2023 |  |  |  |
| --- | --- | --- | --- |
| Treatment | Insecticide | Weed control autumn | Weed control spring |
| A | 0,06L/ha Nexide^1^ against CSFB (2022-09-30) | 2,0 L/ha Focus Ultra^2^ + 0,5 L/ha Belkar^3^+ 1,2 L/ha Kerb^4^ (2022-09-30) | |
| B | 0,06L/ha Nexide^1^ against CSFB (2022-09-30) | 2,0 L/ha Focus Ultra^2^ + 0,5 L/ha Belkar^3^+ 1,2 L/ha Kerb^4^ (2022-09-30) | |
| C |  | row hoeing (2022-10-06) |  |
| D | 0,06L/ha Nexide^1^ against CSFB (2022-09-30) | 2,0 L/ha Focus Ultra^2^ (2022-09-30) |  |
| E/F |  |  |  |
| G |  | row hoeing (2022-09-07) |  |
| H | 0,06L/ha Nexide^1^ against CSFB (2022-09-30) | 2,0 L/ha Focus Ultra^2^ (2022-09-30) |  |
| I |  |  |  |
| J |  | row hoeing (2022-09-07) |  |
| P | 0,06L/ha Nexide^1^ against CSFB (2022-09-30) | 2,0 L/ha Focus Ultra^2^ (2022-09-30)/ row hoeing (2022-10-06) | |
| Q |  | row hoeing (2022-10-06) |  |
| R |  | row hoeing (2022-10-06) |  |
| 2023-2024 |  |  |  |
| A |  | 0,5 Lha Belkar^3^ (2023-10-27) | 165g/ha Matrigon^5^ (2024-03-26) |
| B |  | 0,5 L/ha Belkar^3^ (2023-10-27) | 165g/ha Matrigon^5^ (2024-03-26) |
| C |  | row hoeing (2023-09-18) | row hoeing (2024-03-19) |
| D |  |  | 165g/ha Matrigon^5^ (2024-03-26) |
| E |  |  |  |
| F |  |  | row hoeing (2024-03-19) |
| G |  |  | row hoeing (2024-03-19) |
| H |  |  | 165g/ha Matrigon^5^ (2024-03-26) |
| I |  |  | row hoeing (2024-03-19) |
| J |  |  | row hoeing (2024-03-19) |
| P |  | row hoeing (2023-09-18) |  |
| Q/R |  |  |  |

**Fig S1.** The average number of winter oilseed rape (WOSR) plants per m^2^ in April over two years, 2023 (triangles) and 2024 (dots) in respect to cropping system (oilseed rape sole crop, or intercropped with either berseem clover, spring faba bean or winter pea. Shown are data points (shaded), estimated marginal means and 95% confidence intervals. Different letters denote treatments that are significantly different from each other (p<0.05) based on pairwise contrasts with Tukey correction.

|  | **Fig S2.** Cabbage stem flea beetle larvae per plant, winter oilseed rape (WOSR) fresh plant weight and stem diameter in April over two years, 2023 (triangles) and 2024 (dots). a) Average number of cabbage stem flea beetle (CSFB) larvae per WOSR plant in respect to cropping system (WOSR sole crop, or intercropped with either berseem clover, spring faba bean or winter pea) and input level (high, low). WOSR fresh plant weight (g) in respect to b) cropping system, c) sowing delay of the intercrop (no delay, 10 days or 6 weeks delay) and d) WOSR row distance (narrow: 12.5 cm; wide: 50 cm). WOSR stem diameter (mm) in respect to e) sowing delay of the intercrop and f) cropping system. Shown are data points (shaded), estimated marginal means and 95% confidence intervals. Different letters denote treatments that are significantly different from each other (p<0.05) based on pairwise contrasts with Tukey correction. |
| --- | --- |
|  |  |

**Fig S3.** The rate of parasitism of cabbage seed weevil larvae in 2024 in respect to a) sowing delay of the intercrop and b) and interaction between input level and cropping system (winter oilseed rape sole crop, or intercropped with either berseem clover, spring faba bean or winter pea) and input level (high, low). Shown are data points (shaded), estimated marginal means and 95% confidence intervals. Different letters denote treatments that are significantly different from each other (p<0.05) based on pairwise contrasts with Tukey correction.

**Fig S4.** The average winter oilseed rape (WOSR) seed yield (kg per ha, 9% moisture content) in respect to cropping system (WOSR sole crop, or intercropped with either berseem clover, spring faba bean or winter pea) and input level (high, low) over two years, 2023 (triangles) and 2024 (circles). Shown are data points (shaded), estimated marginal means and 95% confidence intervals. Different letters denote treatments that are significantly different from each other (p<0.05) based on pairwise contrasts with Tukey correction.
